# Catalytic oxidation of small organic molecules by cold plasma in solution in the presence of molecular iron complexes^†^

**DOI:** 10.1038/s41598-020-78683-7

**Published:** 2020-12-10

**Authors:** Dariusz Śmiłowicz, Friederike Kogelheide, Anna Lena Schöne, Katharina Stapelmann, Peter Awakowicz, Nils Metzler-Nolte

**Affiliations:** 1 Inorganic Chemistry I – Bioinorganic Chemistry, Ruhr University Bochum, 44780 Bochum, Germany; 2 Institute for Electrical Engineering and Plasma Technology, Ruhr University Bochum, 44780 Bochum, Germany; 3 Department of Nuclear Engineering, North Carolina State University, Raleigh, NC 27695 USA

**Keywords:** Coordination chemistry, Catalysis, Plasma physics

## Abstract

The plasma-mediated decomposition of volatile organic compounds has previously been investigated in the gas phase with metal oxides as heterogeneous catalysts. While the reactive species in plasma itself are well investigated, very little is known about the influence of metal catalysts in solution. Here, we present initial investigations on the time-dependent plasma-supported oxidation of benzyl alcohol, benzaldehyde and phenol in the presence of molecular iron complexes *in solution*. Products were identified by HPLC, ESI-MS, FT-IR, and $^{1}\hbox {H NMR}$ spectroscopy. Compared to metal-free oxidation of the substrates, which is caused by reactive oxygen species and leads to a mixture of products, the metal-mediated reactions lead to one product cleanly, and faster than in the metal-free reactions. Most noteworthy, even catalytic amounts of metal complexes induce these clean transformations. The findings described here bear important implications for plasma-supported industrial waste transformations, as well as for plasma-mediated applications in biomedicine, given the fact that iron is the most abundant redox-active metal in the human body.

## Introduction

**Figure 1:**
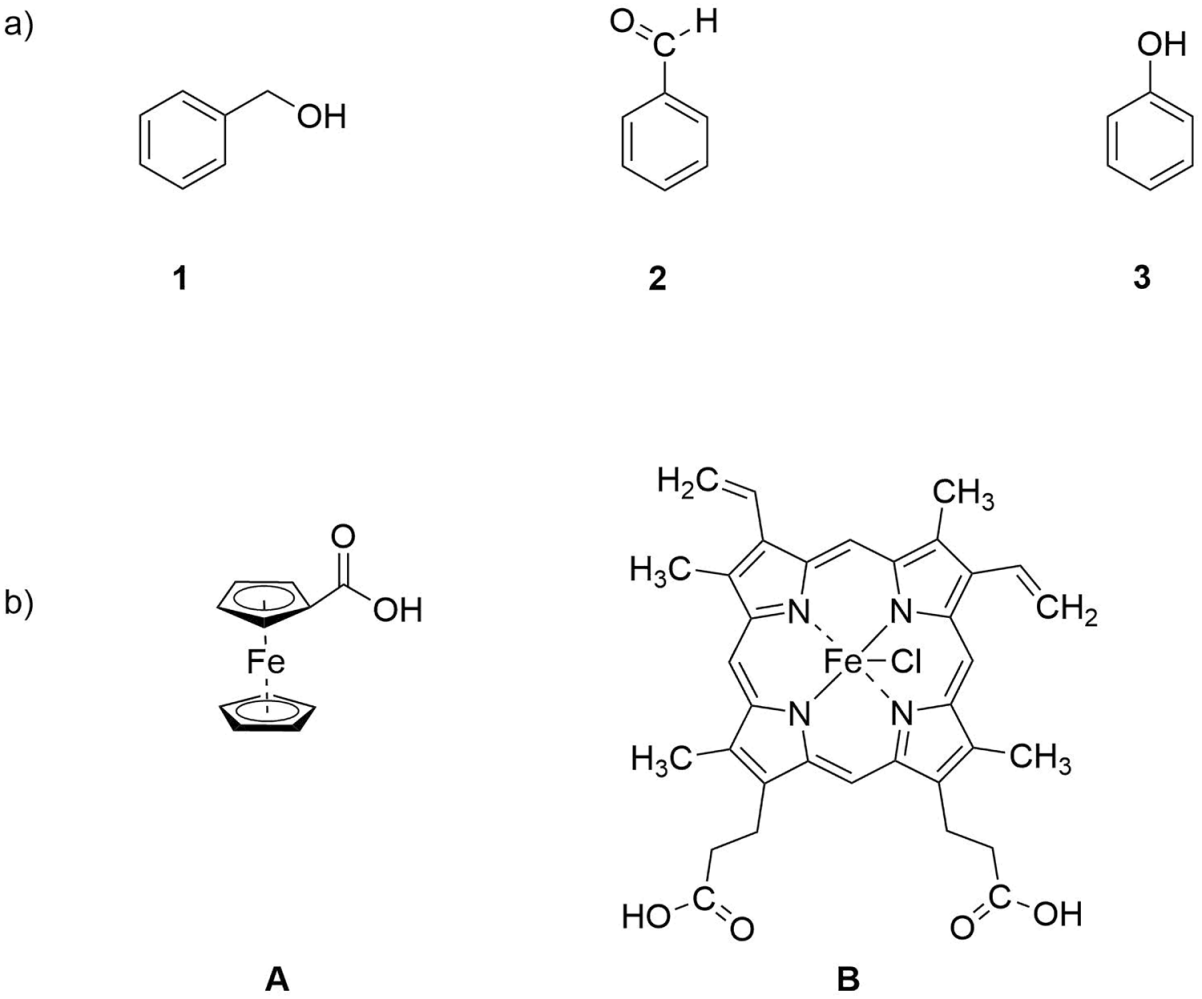
Structures of (**a**) substrates and (**b**) iron complexes.

The chemical and pharmaceutical industries produce a large amount of wastes and pollutants^1–3^. The selective removal or conversion of air and water pollutants consisting of volatile organic compounds (VOCs) constitutes one of the biggest challenge in modern manufacturing^4–6^. Recently, treatment by non-thermal dielectric-barrier discharge (DBD) plasma has received some attention in waste management to provide selective oxidation of hazardous, organic compounds in the chemical industry^7–9^. Since DBD plasma generates vast numbers of reactive species, such as reactive nitrogen and oxygen species, ozone and hydrogen peroxide, it has been utilized to convert toxic compounds to non-hazardous oxidation products^10–12^. So far, heterogeneous catalysis has been involved to neutralize volatile organic compounds^13–15^. Small organic molecules like phenol, benzene, toluene, butyraldehyde and triclocarban were treated with DBD plasma in the presence of metal-oxides as catalysts, including $\hbox {TiO}_{2}$, $\hbox {MnO}_{2}$, $\hbox {Fe}_{2} \hbox {O}_{3}$ and $\hbox {CeO}_{2}$^16–18^. Unfortunately, this resulted in low percentage of conversion only, and numerous oxidation by-products^19,20^. Recently, we investigated the time-dependent chemical modifications of glutathione (GSH) and oxidized glutathione dimer (GSSG) caused by DBD plasma under ambient, aqueous conditions in the presence and absence of iron(II) and iron(III) complexes. We showed that involving either iron(II) or iron(III) complexes in stoichiometric amounts resulted in one clean main oxidation product, namely glutathione sulfonic acid ($\hbox {GSO}_{3}\hbox {H}$)^21^. These results are in contrast to results gained when GSH / GSSG was treated with a DBD plasma alone, which gave a broad mixture of products^22,23^. Encouraged by those previous results, we decided to explore the plasma-assisted oxidation of small molecules under ambient conditions in solution, and in the presence of the same stable iron compounds as homogeneous catalysts, namely ferrocenecarboxylic acid (**A**) and hemin (**B**, Fig. 1). In exploratory work we describe in this communication the treatment of organic model compounds (benzyl alcohol, benzaldehyde and phenol, Fig. 1) in aqueous solutions containing iron complexes **A** and **B** with a dielectric barrier discharge (DBD) plasma.

DBDs are known to generate a wide range of reactive species, such as ozone or nitric oxide^24–26^, depending on the exact conditions^27,28^. A standardized DBD was used in this work, which was already extensively characterised by us in previous studies regarding its plasma parameters, such as the electron density or the gas temperature, and the generated concentration of reactive chemical species^29,30^. Among others, the ozone density amounts to $7 \times 10^{16}\,\hbox {cm}^{-3}$ and the measured NO density is equal to $1.2 \times 10^{15}\,\hbox {cm}^{-3}$ for the operation parameters used in this study^30^. Moreover, optical emission spectroscopy (OES) was applied to measure the UV irradiance emitted by the DBD. The UV-A and UV-B irradiance amounts to $1.1 \times 10^{-4}\,\hbox {J}\,\hbox {s}^{-1}\hbox {cm}^{-2}$ and $1.1 \times 10^{-5}\,\hbox {J}\,\hbox {s}^{-1}\hbox {cm}^{-2}$, respectively^30^. The present studies were carried out under the same experimental conditions, namely under ambient air, at room temperature and at atmospheric pressure.

## Results

**Figure 2:**
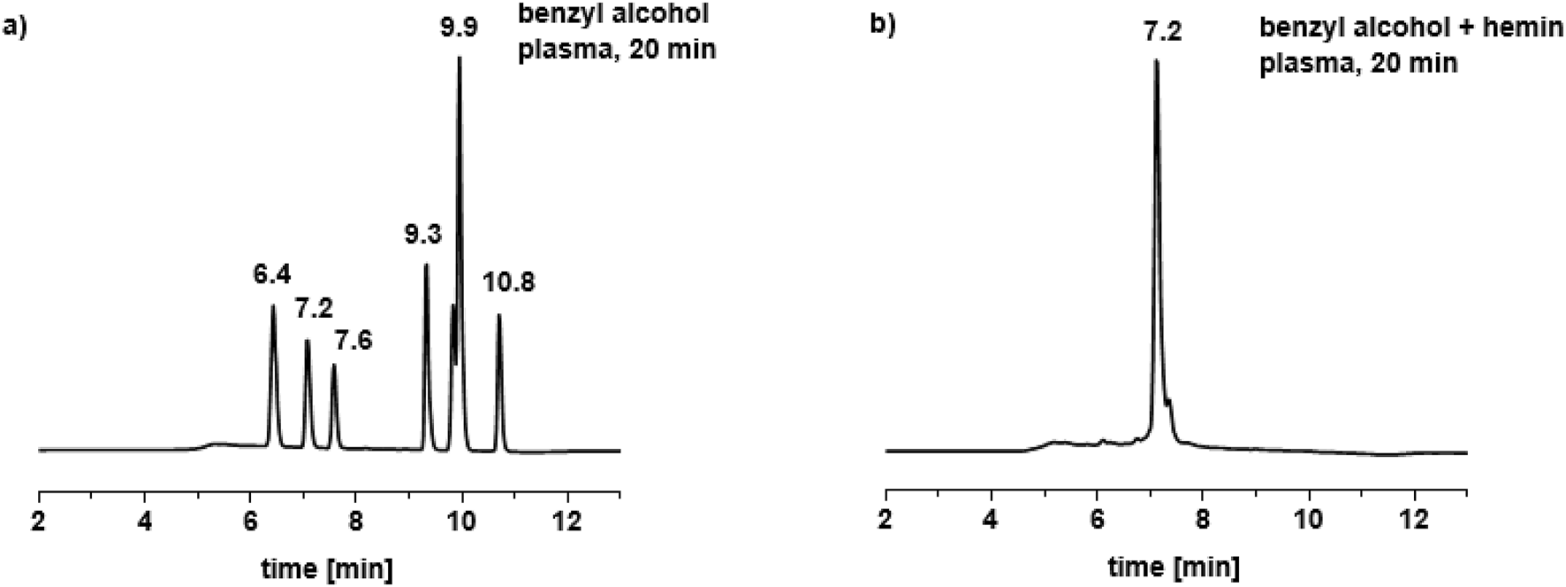
HPLC trace of benzyl alcohol after plasma treatment (**a**) without metal complex, (**b**) in the presence of complex **B**.

In a first step, the time-dependent impact of DBD treatment on the organic molecules alone, i.e. without iron complexes was investigated. Benzyl alcohol (compound **1**), benzaldehyde (compound **2**) and phenol (compound **3**) were treated with plasma for 1, 3, 5 and 20 min in aqueous solution. Treatment of **1** with the DBD for 1 min revealed a mixture of products according the HPLC trace of the solution. Extending the treatment time to 3, 5 min as well as to 20 min caused no further changes in the distribution of products (Fig. 2a).

**Figure 3:**
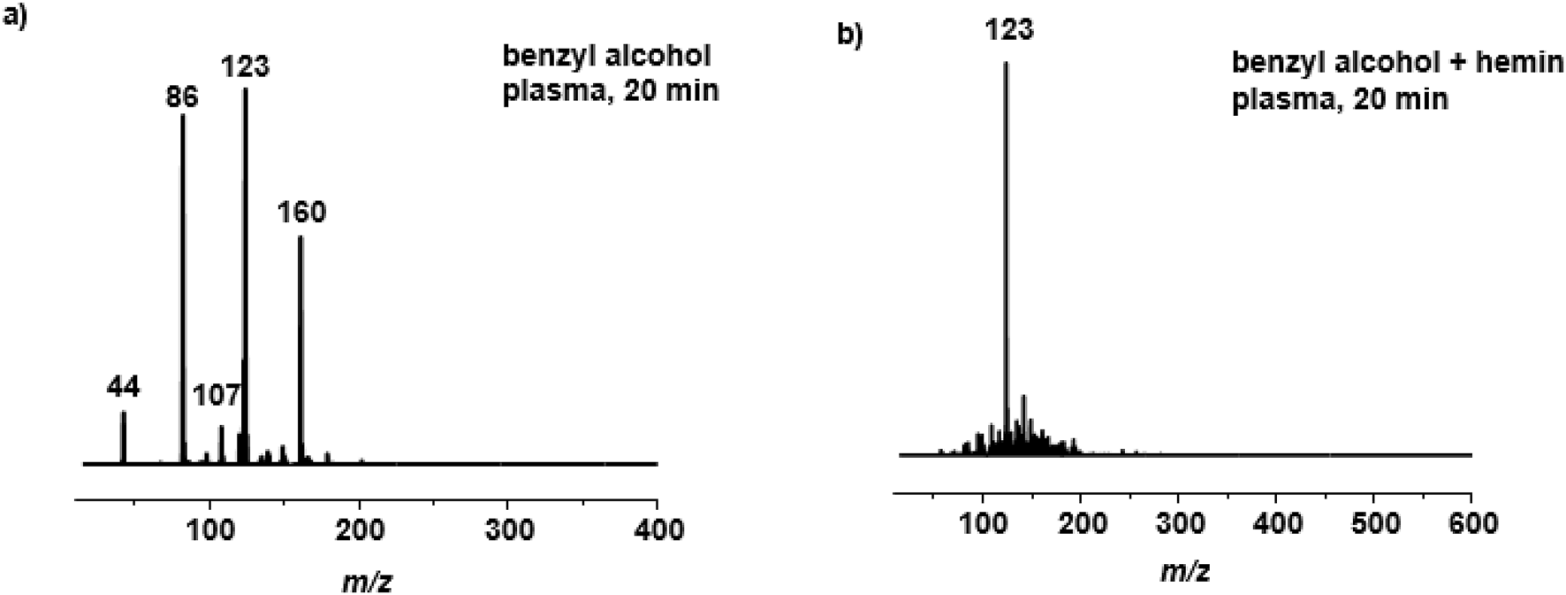
ESI-MS spectrum of benzyl alcohol after plasma treatment (**a**) without metal complex, (**b**) in the presence of complex **B**.

Among the mixture of products, we were able to identify four compounds by mass spectrometry (signals in the ESI-MS at $\textit{m/z} = 44$, 86, 107 and 123). All signals correspond to $[\hbox {M}{+}\hbox {H}]^{+}$ species (Fig. 3a) and they were assigned to propene, hexa-2,4-dien, benzaldehyde and benzoic acid, respectively (Fig. 5a). Compound **2**, when treated with plasma, displayed the same products after 1, 3, 5 and 20 min. When compound **3** was treated with plasma alone, the resulting HPLC and ESI-MS data showed a mixture of products (phenol, biphenyl, 2,2’-biphenol, benzoic acid), mostly the same as those observed for benzene oxidation using DBD by Dey et al.^31^.

In the next step, the impact of plasma on compounds **1**, **2** and **3** in the presence of a stoichiometric amount of ferrocenecarboxylic acid (complex **A**) and hemin (complex **B**) was investigated. Both iron complexes were chosen for this work because they exhibit high stability in aqueous medium during plasma treatment, as shown in a previous study^21^. They differ however in the oxidation state of iron, with **A** being Fe(+II), and the Fe center in hemin (**B**) being +III. For benzyl alcohol after 1 and 3 min of plasma treatment in the presence of iron(III) complex **B** only two compounds were detected, namely benzaldehyde and benzoic acid (Fig. 5b). However, after 5 min, benzoic acid was observed as the only species, indicating a complete oxidation of the benzylic group, whereas without iron complex, benzoic acid constitutes only 8 % of all final products (Fig. 2a). Extending the plasma treatment time to 20 min did not influence the number or structure of products. Also, treatment of **1** with plasma in the presence of **A** resulted largely in the same products. The data are available in the $\hbox {ESI}\dag$. The HPLC profile after 20 min of plasma treatment in the presence of both iron complexes showed one signal at 7.2 min (cf. Fig. 2b) with *m/z* value at 123 on ESI-MS spectrum (Fig. 3b). The same oxidation pattern was observed in the case of benzaldehyde. After 1 min of plasma treatment with complexes **A** or **B** two compounds were observed according to HPLC and ESI-MS analysis, namely benzaldehyde and benzoic acid. After 3, 5 and 20 min only benzoic acid was observed as the sole oxidation product. However, in the case of phenol, several products with functional groups were observed (benzaldehyde, benzoic acid), among many products after 20 min of plasma treatment ($\hbox {ESI}\dag$).

**Figure 4:**
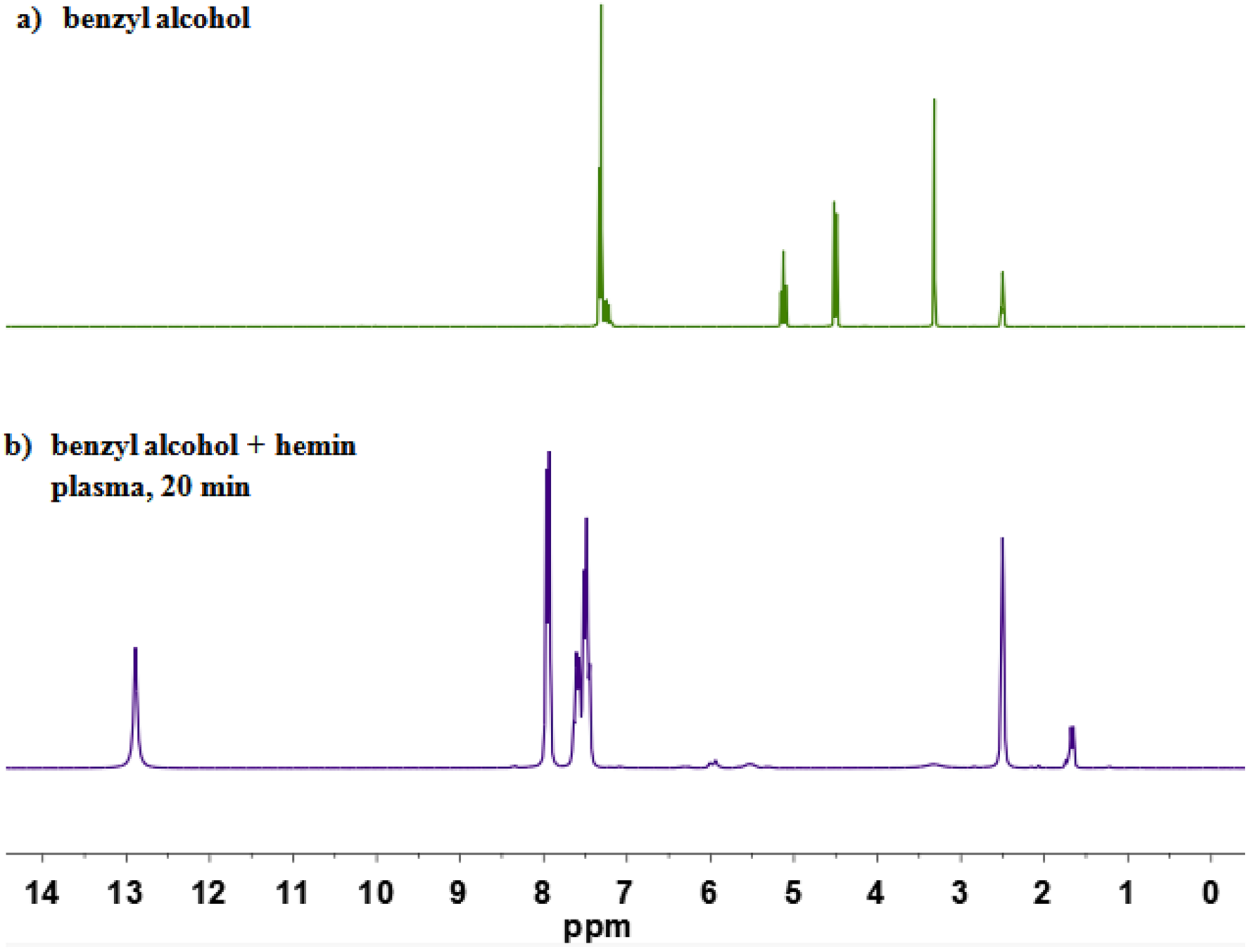
$^{1}\hbox {H NMR}$ spectrum of (**a**) benzyl alcohol, (**b**) benzyl alcohol after 20 min of plasma treatment in the presence of complex **B** (both spectra recorded in DMSO-d6).

FT-IR spectroscopy confirmed the presence of the carboxylic acid group after plasma treatment with a peak at $1685\,\hbox {cm}^{-1}$ for the $\hbox {C}{=}\hbox {O}$ stretching vibration ($\hbox {ESI}\dag$), which agrees with the characteristic bands for benzoic acid in literature^32^. $^{1}\hbox {H NMR}$ spectroscopy ultimately confirmed nature of the product after oxidation of substrates by DBD plasma treatment (Fig. 4) by showing the protons from the aromatic ring at 7.45–7.96 ppm and a signal at 12.88 ppm corresponding to the OH from the carboxylic acid group of benzoic acid. Notably, the absence of an aldehyde proton around 10 ppm indicates complete conversion with no intermediate oxidation product left. Also notably, the clean $^{1}\hbox {H NMR}$ spectrum excludes any further oxidation or degradation of benzoic acid.

**Figure 5:**
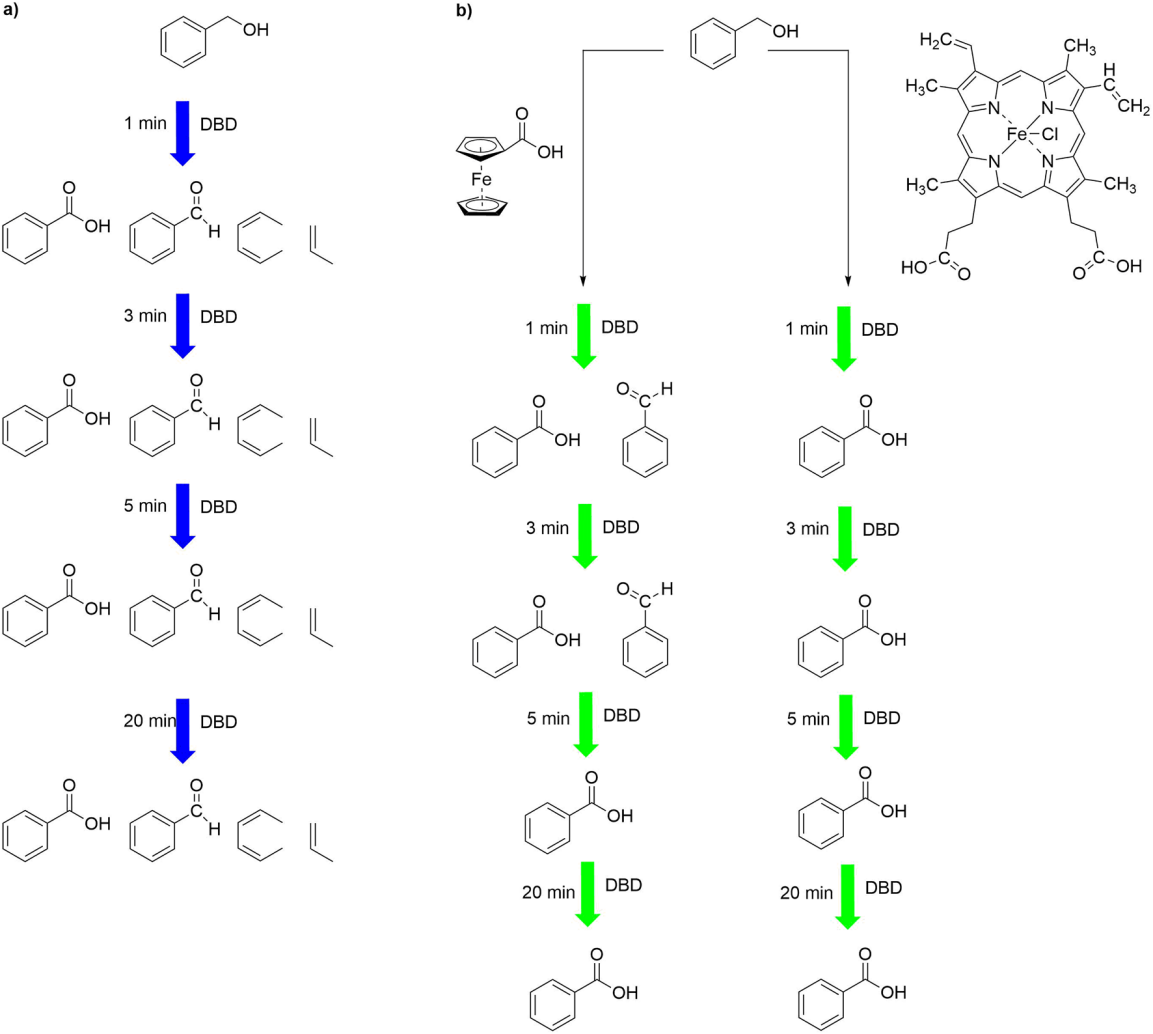
Chemical modifications of benzyl alcohol after different times of plasma treatment (**a**) without iron complexes, (**b**) in the presence of iron complexes.

In order to establish whether these rather clean conversions were stoichiometric in nature the DBD-supported oxidation was carried out in the presence of 10 % of the iron complexes. For all substrates the oxidation pattern appeared to be identical, with benzoic acid as main oxidation product, in comparison to experiments carried out with the stoichiometric amount ($\hbox {ESI}\dag$). According to the results (cf. Fig. 5), the oxidation products do not depend on the nature of iron complexes, i.e. the Fe(II) and Fe(III) complexes give an identical spectrum of products, regardless of the oxidation state of the Fe center. Only small time-dependent differences can be observed since the oxidation to the final product is accomplished faster in the presence of hemin, the iron(III) complex.

To complete the picture, two sets of control experiments were carried out: Stability experiments in distilled water for molecules **1**, **2** and **3** alone, and interaction studies of **1**, **2** and **3** with both iron(II) and iron(III) complexes, all investigated by HPLC and ESI-MS. All organic substrates were completely stable after 1, 3, 5 and 20 min, making them suitable candidates for plasma investigations. The HPLC traces and the full-scan mass spectra are available in the $\hbox {ESI}\dag$. The iron complexes were incubated with compounds **1**, **2** and **3** in aqueous solution for 1, 3, 5 and 20 min in the ratio 1:1, as well as with the substoichiometric amount (10 %) of iron complexes. During the whole incubation time no modification of the organic molecules nor of the iron complexes was observed. The stability of the iron complexes in aqueous solution as well as the impact of plasma on those complexes was investigated by us previously^21^. Both iron(II) and iron(III) complexes exhibited high stability in aqueous medium during plasma treatment with no traces of decomposition, which makes them suitable candidates for the above studies.

Since both iron complexes possess pendant carboxylate groups, additional control experiments were carried out using benzoic acid as the catalyst to prove that the iron centre and not a carboxylate unit per se is responsible for the observed oxidation results. Benzyl alcohol, benzaldehyde and phenol were treated by cold plasma in the presence of a stoichiometric amount of benzoic acid for 1, 3 and 5 min. HPLC and ESI-MS analyses revealed oxidation patterns similar to those obtained from the experiments without iron (II) and iron (III) complexes. At the same time, benzoic acid appeared to be stable during plasma treatment in the absence of metal complexes ($\hbox {ESI}\dag$).

## Discussion

In summary, we report here the use of iron complexes as catalysts for the specific transformation of organic substrates under the influence of cold, atmospheric pressure plasma. Degradation as well as a whole range of oxidation products were observed for compounds **1**, **2** and **3** after DBD plasma treatment alone, while only one oxidation product was more rapidly found after plasma treatment in the presence of stoichiometric as well as substoichiometric (10 %) amounts of the iron complexes **A** and **B**. Chromatography as well as spectroscopic analysis reveal that benzoic acid is the main oxidation product after Fe-assisted plasma treatment of benzyl alcohol and benzaldehyde under ambient air, at room temperature and at atmospheric pressure. Ferrocenecarboxylic acid (with Fe(II)) as well as the Fe(III) hemin complex are suitable for homogeneous oxidation of these organic compounds by DBD plasma, since both species are redox active. Details of how these metal complexes interact with the reactive species initially generated by the plasma remain elusive at these early stages of investigation, but general chemical wisdom would suggest Fenton-type and / or dismutase-like chemistry involving redox cycling between Fe(II) and Fe(III) species. This would also explain why compounds **A** and **B** are more or less equally active in our experiments, irrespective of the different oxidation states of the iron complexes originally. What is crucial however is to use stable iron complexes, because as we reported previously, some iron(III) complexes, for example iron(III) acetylacetonate undergo decomposition during plasma treatment and further interact with substrates during plasma treatment^21^.

The findings of this investigation complement those of earlier studies involving DBD plasma for oxidation assistance. Nevertheless, two important new aspects emerge from our investigations above. First, metal complexes — as exemplified by redox-active iron compounds in this study — are able to work in concert with plasma-generated species, like ROS, and they do so even at catalytic amounts. The metal complexes will not necessarily make the oxidation reaction faster, but can drive the reaction towards a more uniform product spectrum. This may be highly desirable for industrial applications, such as transformation of organic waste products from an industrial process to one, possible value-added product rather than a product mixture that must be considered waste.

The second important conclusion from this work concerns the application of cold plasma in biology and medicine, e.g. in wound healing^33,34^. So far, reactive species like ROS and RNS were considered responsible for triggering the biological responses. However, metals are almost ubiquitous in biology as cofactors in metalloenzymes, and iron in particular is by far the most abundant redox-active metal in the human body, prominently present in hemoglobin. It is highly likely that metal-containing biomolecules, like hemoglobin, play an important role in mediating the presence and nature of plasma-generated species, and will thereby have a decisive influence on the biological outcome of plasma-triggered medical treatments. This aspect has not been considered in previous work at all. It will be the focus of future investigations from our groups.

## Materials and methods

### Experimental setup

The experimental setup used in this work has been described in a similar way before^21,22^. Briefly, the experiments in this study were carried out with a dielectric barrier discharge, which consists of a copper electrode covered with aluminium oxide ($\hbox {Al}_{2} \hbox {O}_{3}$) with a thickness of 1 mm. The electrode has a diameter of 10 mm and the distance between the driven electrode and the sample was kept constant at 1 mm. The samples were placed on a grounded aluminium plate and ambient air was used as the process gas. The temperature in the lab was adjusted to 20 $^{\circ }\hbox {C}$ and the relative humidity varied between 40 and 50 % during the period in which the experiments were carried out. The experimental setup is described in more detail in Kogelheide et al. and a scheme of the dielectric barrier discharge can be found in the ESI, Scheme $\hbox {S1}^\dag$^35^. The electrode was driven with a pulsed power supply^29^. For the experiments in this study the repetition frequency was set to 300 Hz and the amplitude of the HV pulse to $24\,\hbox {kV}_{pp}$. The dielectric barrier discharge has been characterised regarding several plasma parameters as well as reactive species. In Kogelheide et al., the electron density distribution in the discharge is described in detail^29^. The radial profiles of the plasma produced oxygen species, atomic oxygen (O) and ozone ($\hbox {O}_3$), within the plasma volume of the former used plasma source are determined using two-photon laser-induced fluorescence spectroscopy (TALIF) and optical absorption spectroscopy (OAS) in Baldus et al. Furthermore, a model of the afterglow chemistry is described in this paper to obtain insight into the dynamics of the considered reactive oxygen species^36^.

### Materials

All reagents and chemicals were purchased from commercial sources and used without further purification. Ferrocenecarboxylic acid was purchased from Abcr. Chloro(protoporphyrinato)iron(III) (hemin), benzyl alcohol, benzaldehyde, phenol and N,N-diisopropylethylamine (DIEA) were purchased from Sigma-Aldrich.

### Samples preparation

A similar protocol for sample treatment was published by us previously^21^. Small organic molecules were dissolved in distilled water with a concentration of 4 mg/ml. Ferrocenecarboxylic acid and chloro(protoporphyrinato)iron(III) (hemin) were dissolved in distilled water with a concentration of 4 mg/ml with 2 eq. of DIEA. $10\,\upmu \hbox {l}$ were placed on cleaned silicon wafers (Siltronic AG) and treated with the DBD for 1, 3, 5 and 20 min. After treatment, samples were filled into reagent tubes and evaporated liquid replenished with distilled water to the concentration of 1 mg/mL for the analysis via mass spectrometry and HPLC. The samples for the FTIR spectroscopy were dried by desiccation after the plasma treatment. As controls, another sample was prepared equally, omitting the plasma treatment. Control samples were placed in ambient conditions as the sample treated for the longest time.

### Mass spectrometry

Electron spray ionization (ESI) mass spectra were obtained on an Esquire 6000 mass spectrometer (Bruker). Analysis of samples by ESI-MS follows our standard operating procedure that has been described before^21,22^. Full mass spectra of the investigated ferrocenecarboxylic acid, chloro(protoporphyrinato)iron(III) (hemin), benzyl alcohol, benzaldehyde and phenol were acquired in both negative-ion and positive-ion mode with the spectrometer equipped with an ion-trap analyser. Three samples of $10\,\upmu \hbox {l}$ treated for the same time were pooled and diluted tenfold with acetonitrile for $300\,\upmu \hbox {l}$ with a final concentration of 1 mg/ml. Instrumental parameters were tuned for each sample. The capillary voltage was set in a range of -22 to 25 V, the spray voltage was between 3.00 and 4.50 kV, and a capillary temperature of $180\,^{\circ }\hbox {C}$ was employed. The mass scan range was from *m/z* 50 to 2000 amu, for 20 s scan time. Spectra were acquired using a direct infusion setup with a flow rate of $5\,\upmu \hbox {l/min}$ with a cone voltage of 20 kV. To determine occurring in-source fragments, which increase the sample complexity without yielding significant additional information, MS/MS spectra were acquired using the same conditions with a collision energy ramp between 2.00 and 4.00 eV. Spectra were deconvoluted and a background of ten times noise (500 counts in positive and 5 counts in negative mode) was subtracted before peak annotation. All experiments were performed in triplicates.

### FTIR spectroscopy

Analysis of samples by FTIR follows our standard operating procedure that has been described before^21,22^. A Bruker Vertex FTIR-micro spectrometer was used for the analysis of the samples. FTIR spectra were recorded from 750 to $4000\,\hbox {cm}^{-1}$ with a spectral resolution of $4\,\hbox {cm}^{-1}$. For the FTIR spectroscopy of the investigated ferrocenecarboxylic acid, chloro(protoporphyrinato)iron(III) (hemin), benzyl alcohol, benzaldehyde and phenol, 12 spectra were recorded at different positions of each sample with each spectrum representing 32 accumulated spectra. Background spectra were obtained for every samples due to the ambient measurement conditions to compensate water and carbon dioxide content in air^37^. All recorded transmission spectra, *T*, were converted into absorption spectra, *A*:

1 $$\begin{aligned} A = log\left( \frac{1}{T}\right) . \end{aligned}$$

Absorption spectra were baseline corrected afterwards and normalization of the data was carried out applying the Euclidean norm:

2 $$\begin{aligned} a^{norm}_{k} = \frac{a_{k}}{\sqrt{\sum ^{n}_{k=1}(a_{i})^{2}}}. \end{aligned}$$

Every data point of each spectrum ${a}_k$ of wavenumber *k* is normalized to the square root of the sum of every spectrum data point. All experiments were performed in triplicates.

### HPLC

Analysis of samples by HPLC follows our standard operating procedure that has been described before^21^. An HPLC Knauer system with a quaternary pump and a UV-DAD detector equipped with a Nucleodur C4 ec column (125 mm $\times$ 4 mm, internal diameter $5\,\upmu \hbox {m}$, Macherey-Nagel), was used. HPLC was performed by using two buffer systems (buffer A: $\hbox {H}_2\hbox {O/MeCN/TFA}$, 95:5:0.1, v/v/v; buffer B: $\hbox {MeCN/}\hbox {H}_2\hbox {O/TFA}$, 95:5:0.1, v/v/v) as the mobile phase. Chromatography was performed with a linear gradient conditions of buffer B (100 % in 10 min) from 100 % buffer A with a total run time of 50 min. The flow rate of the mobile phase was 1.0 ml/min. $10\,\upmu \hbox {l}$ of the sample was injected. The column was purged with the mobile phase for 2 min, followed by equilibration for 15 min, and then 15 min were required for sample analysis. Spectral data were collected at detection wavelengths of 214 nm and 254 nm, and finally the collected data were plotted (Fig. S1).

## Supplementary information

Supplementary Information
