# Supplementary material for: Catalytic oxidation of small organic molecules by cold plasma in solution in the presence of molecular iron complexes†
Source: Sci Rep. 2020 Dec 10;10:21652. doi: 10.1038/s41598-020-78683-7 (PMC7728814; doi:10.1038/s41598-020-78683-7)
Supplement: Supplementary file 1 — Supplementary Information [file 41598_2020_78683_MOESM1_ESM.pdf]

**Electronic Supporting Information**

**for**

# Catalytic Oxidation of Small Organic Molecules by Cold Plasma in Solution in the Presence of Molecular Iron Complexes

**by**

Dariusz Śmiłowicz[a], Friederike Kogelheide[b], Anna Lena Schöne[b], Katharina Stapelmann[c], Peter Awakowicz[b], Nils Metzler-Nolte[*,a]

[a] Chair of Inorganic Chemistry I – Bioinorganic Chemistry, Faculty of Chemistry and Biochemistry, Ruhr-University Bochum, Bochum, Germany

[b] Institute for Electrical Engineering and Plasma Technology, Ruhr University Bochum, 44780, Bochum, Germany

[c] Department of Nuclear Engineering, North Carolina State University, Raleigh, North Carolina 27695, USA

**Contents**

1. The scheme of the plasma source p.S2
2. Results of stability experiments of substrates p.S2-S9
3. Influence of plasma on substrates in the presence of iron complexes p.S10-S13
4. FT-IR data of substrates after plasma treatment p.S13-S17
5. Literature p.S17

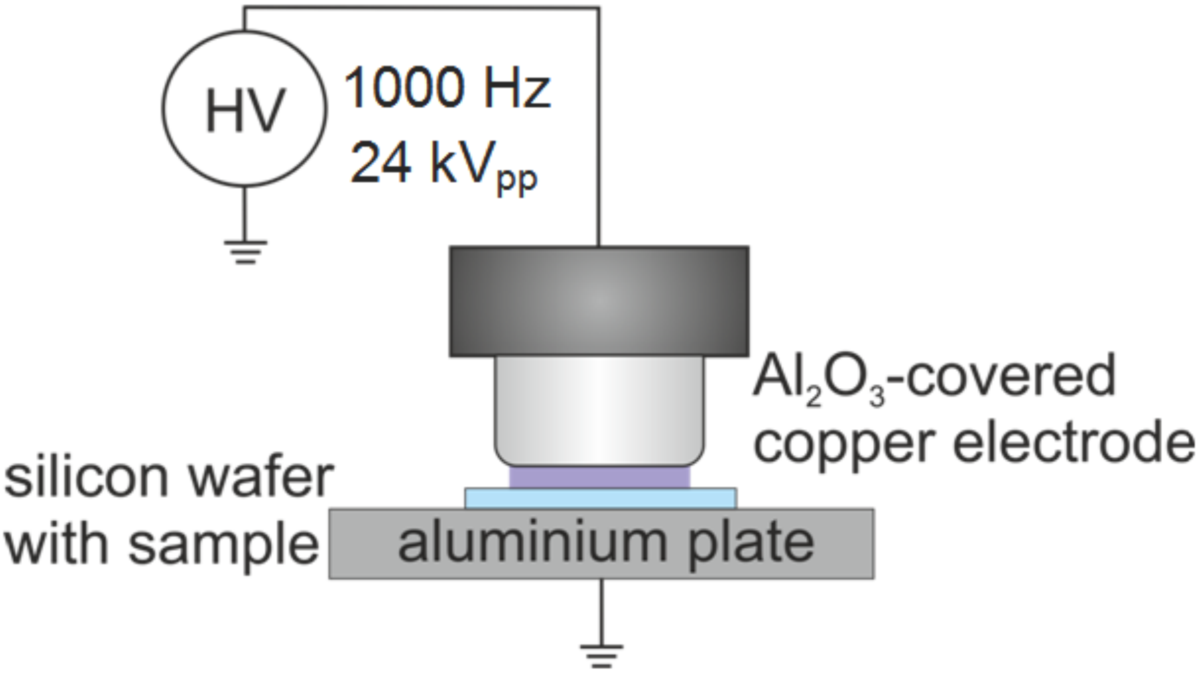


**Figure S1.** The scheme of the plasma source.[1]

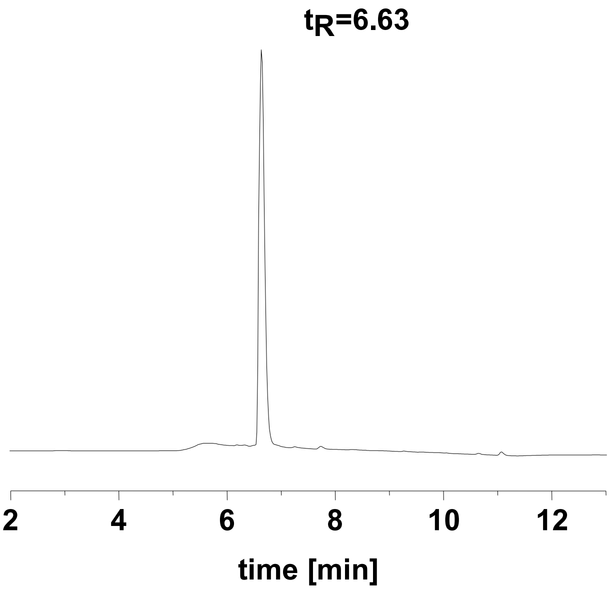


**Figure S2.** HPLC chromatogram of benzyl alcohol (**1**) after 1 min of stability experiments.

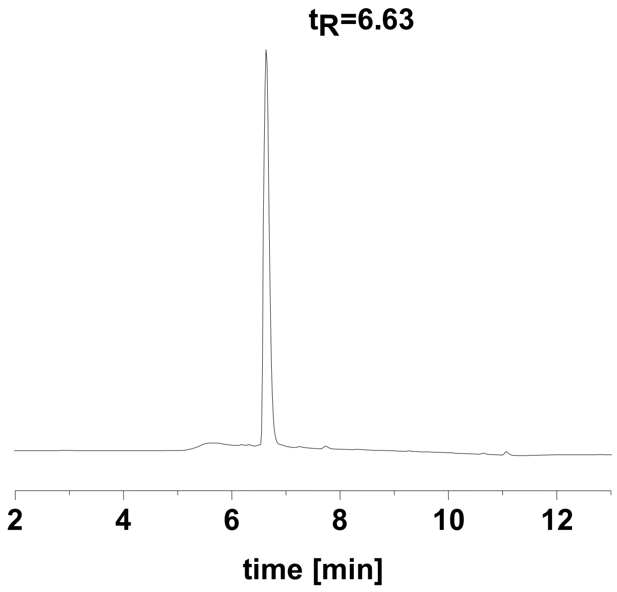


**Figure S3.** HPLC chromatogram of benzyl alcohol (**1**) after 3 min of stability experiments.

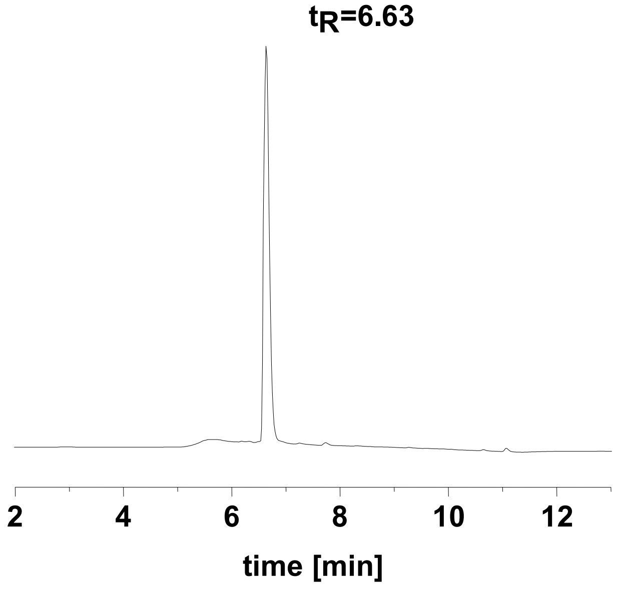


**Figure S4.** HPLC chromatogram of benzyl alcohol (**1**) after 5 min of stability experiments.

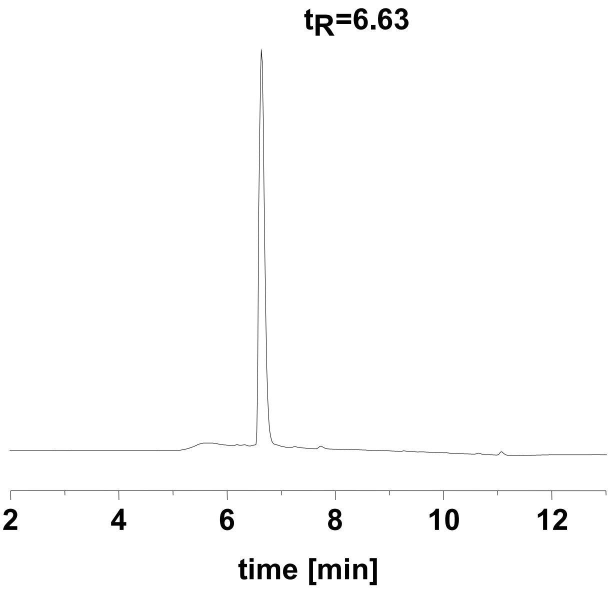


**Figure S5.** HPLC chromatogram of benzyl alcohol (**1**) after 20 min of stability experiments.

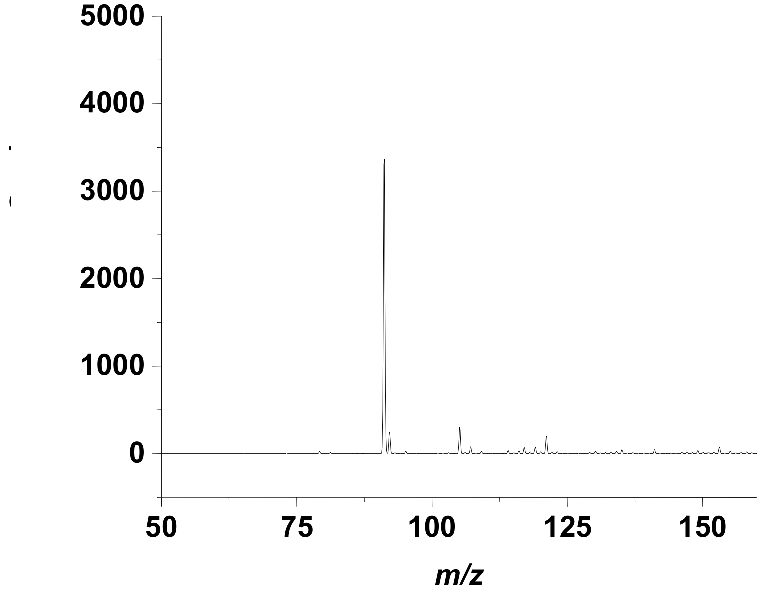


**Figure S6.** ESI-MS spectrum of benzyl alcohol (**1**) after 20 min of stability experiments.

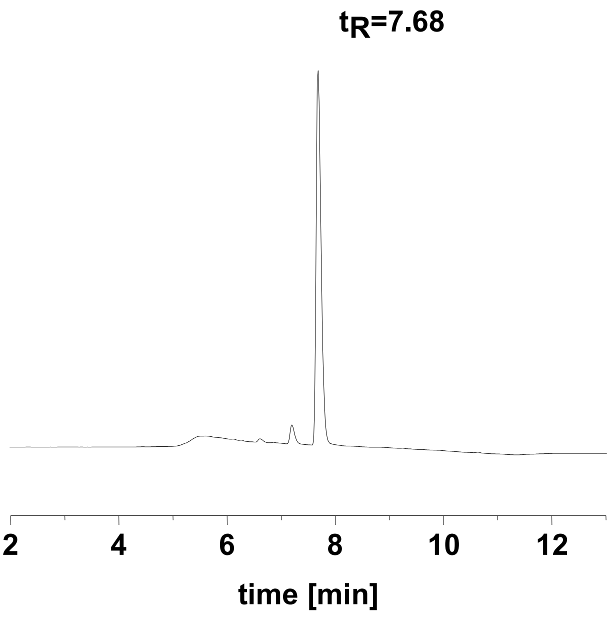


**Figure S7.** HPLC chromatogram of benzaldehyde (**2**) after 1 min of stability experiments.

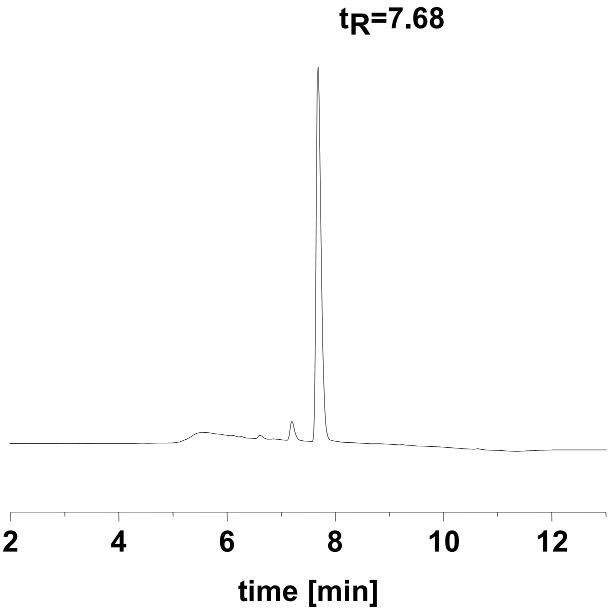


**Figure S8.** HPLC chromatogram of benzaldehyde (**2**) after 3 min of stability experiments.

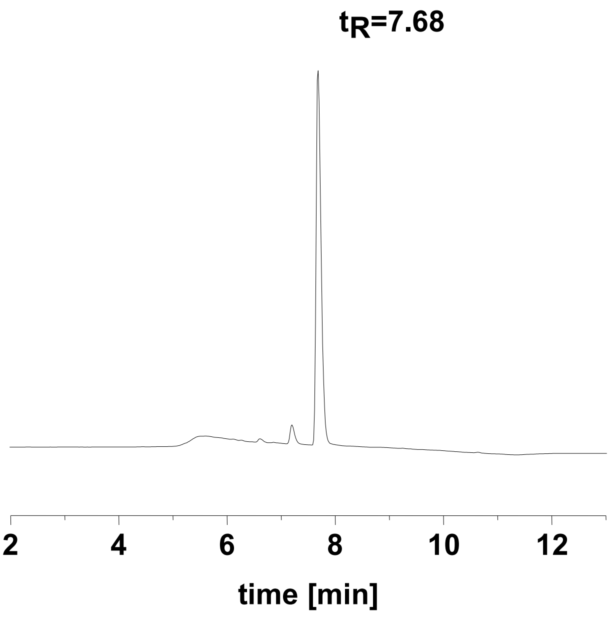


**Figure S9.** HPLC chromatogram of benzaldehyde (**2**) after 5 min of stability experiments.

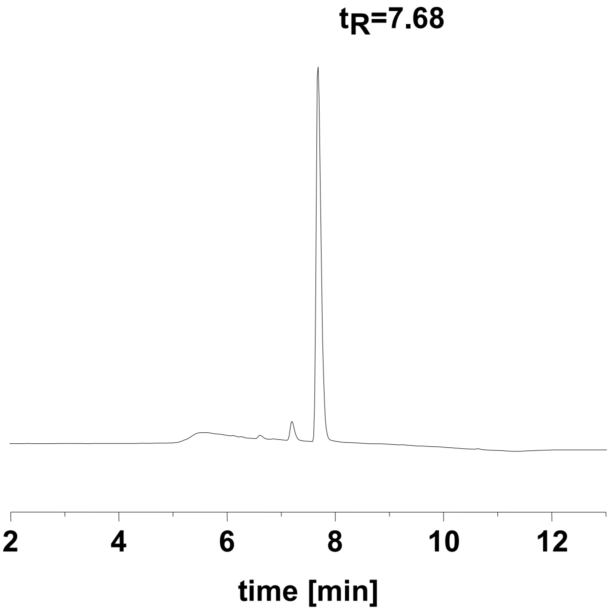


**Figure S10.** HPLC chromatogram of benzaldehyde (**2**) after 20 min of stability experiments.

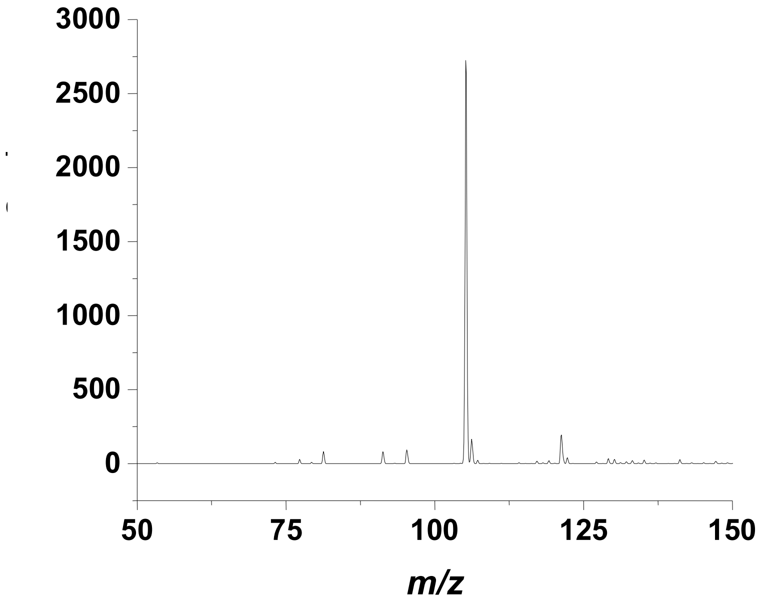


**Figure S11.** ESI-MS spectrum of benzaldehyde (**2**) after 20 min of stability experiments.

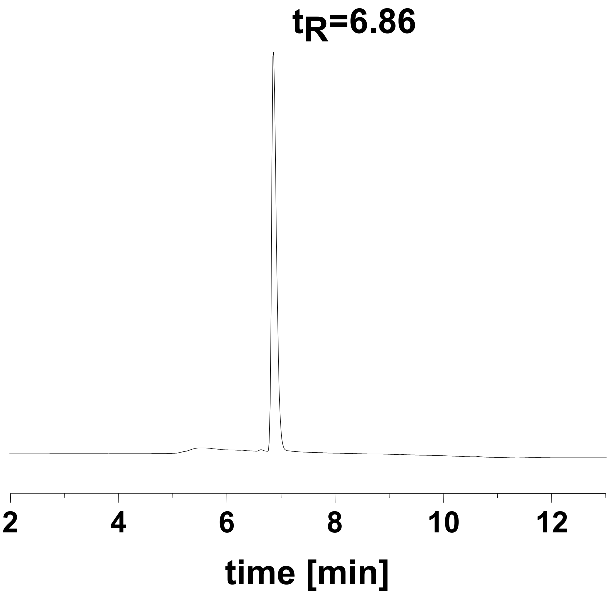


**Figure S12.** HPLC chromatogram of phenol (**3**) after 1 min of stability experiments.

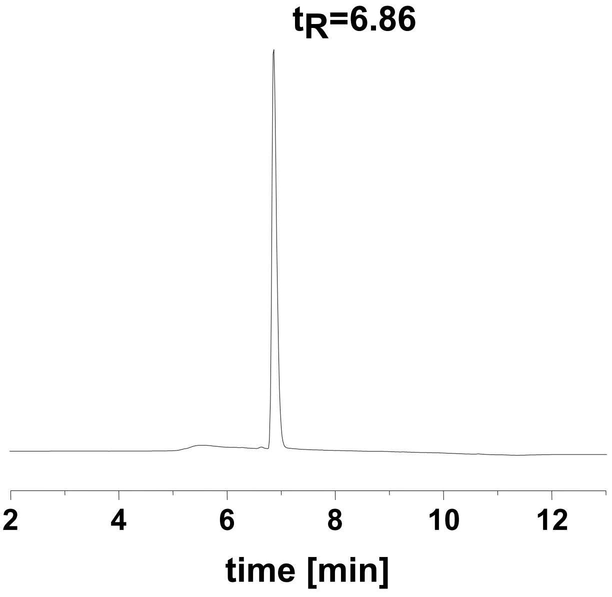


**Figure S13.** HPLC chromatogram of phenol (**3**) after 3 min of stability experiments.

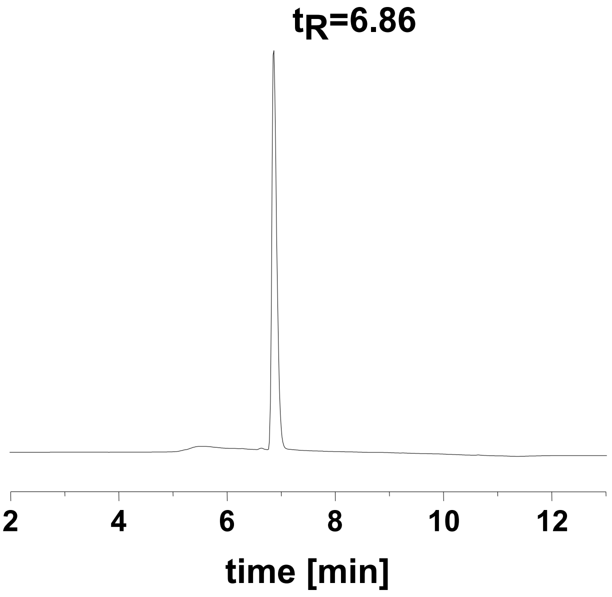


**Figure S14.** HPLC chromatogram of phenol (**3**) after 5 min of stability experiments.

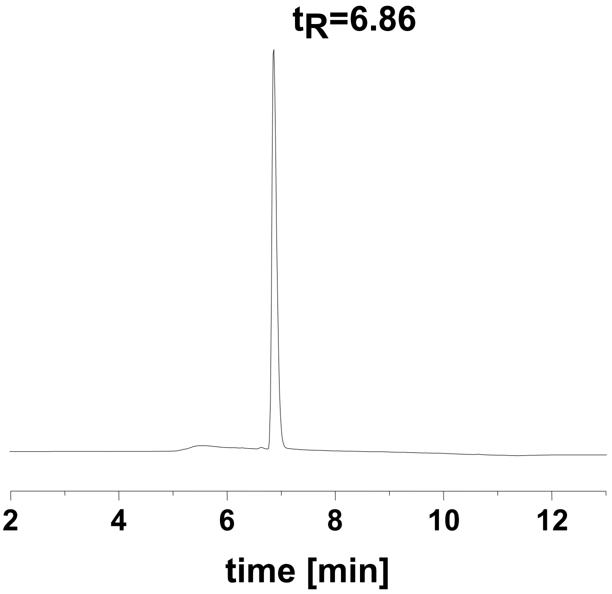


**Figure S15.** HPLC chromatogram of phenol (**3**) after 20 min of stability experiments.

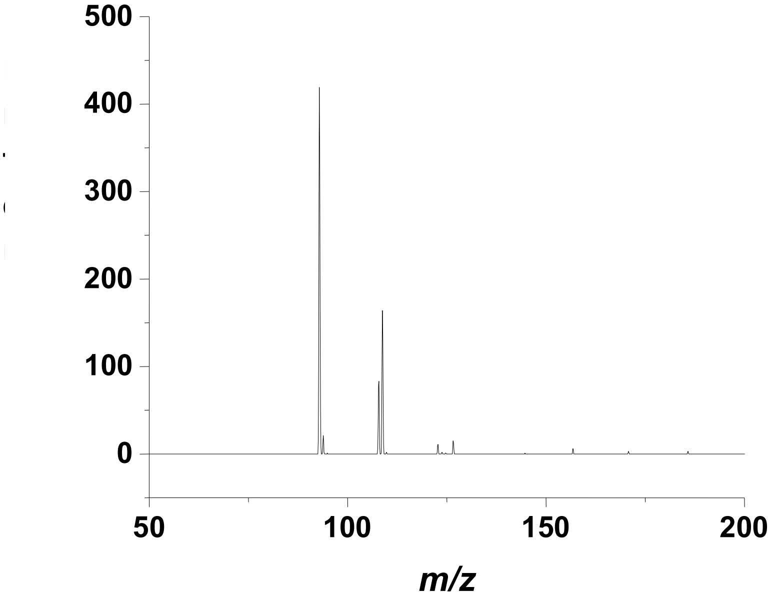


**Figure S16.** ESI-MS spectrum of phenol (**3**) after 20 min of stability experiments.

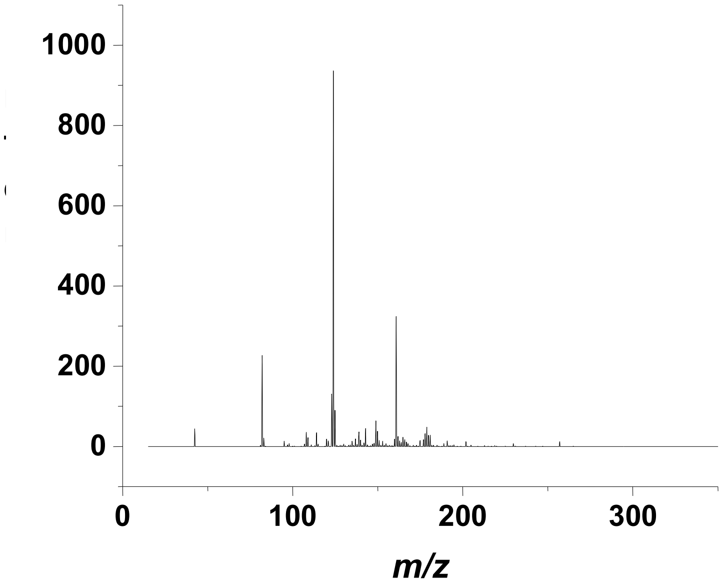


**Figure S17.** ESI-MS spectrum of benzaldehyde after 20 min of plasma treatment without metal complex.

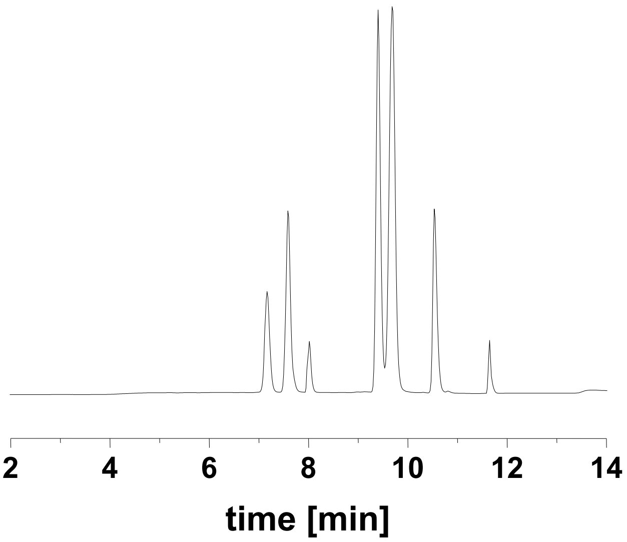


**Figure S18.** HPLC chromatogram of benzaldehyde after 20 min of plasma treatment without metal complex.

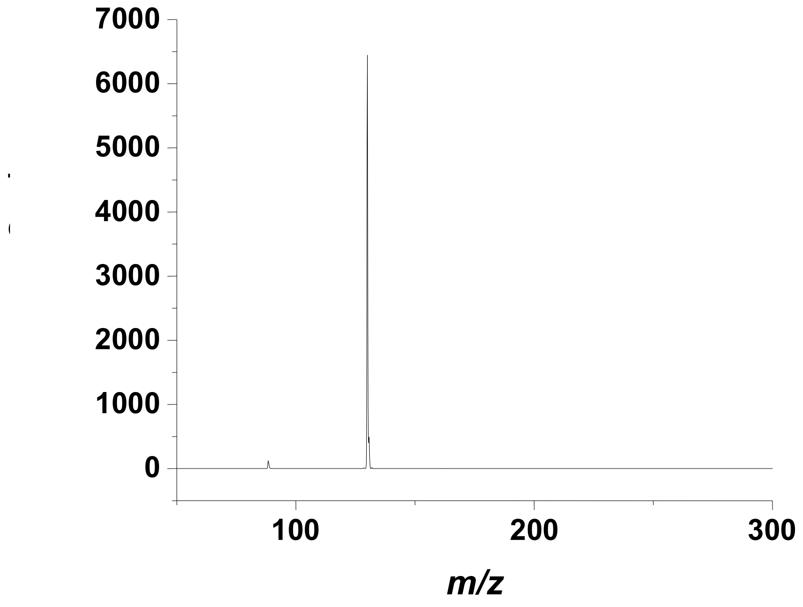


**Figure S19.** ESI-MS spectrum of benzaldehyde after 20 min of plasma treatment in the presence of 10% of the complex **A/B**

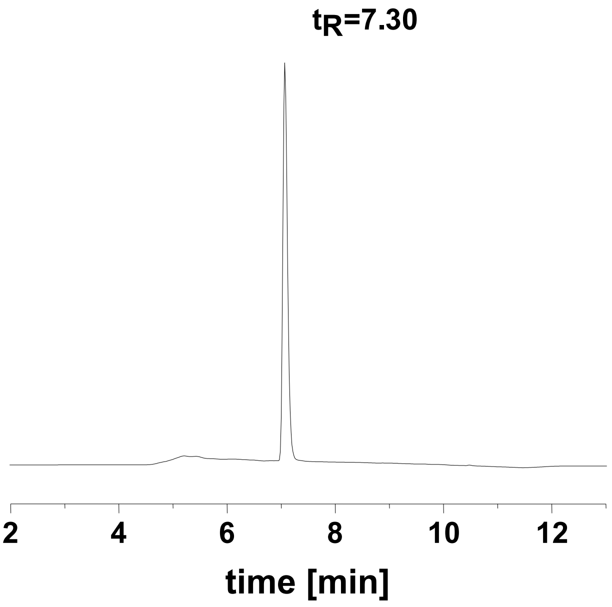


**Figure S20.** HPLC chromatogram of benzaldehyde after 20 min of plasma treatment in the presence of 10% of the complex **A/B**

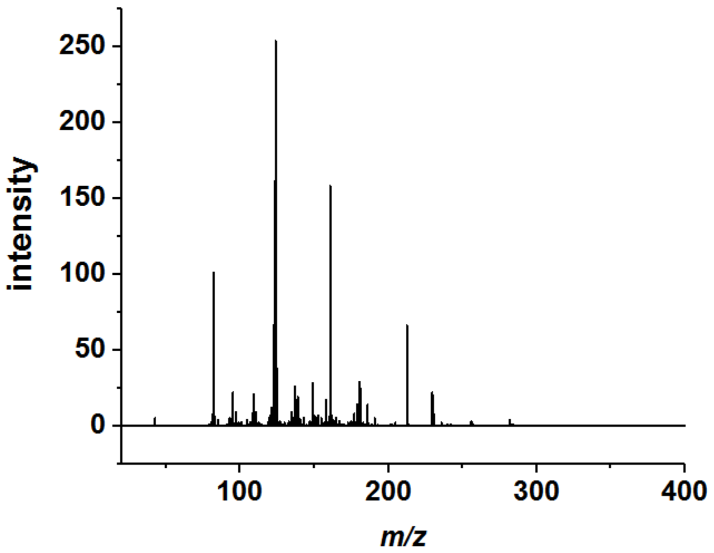


**Figure S21.** ESI-MS spectrum of phenol after 20 min of plasma treatment in the presence of 10% of the complex **A/B**

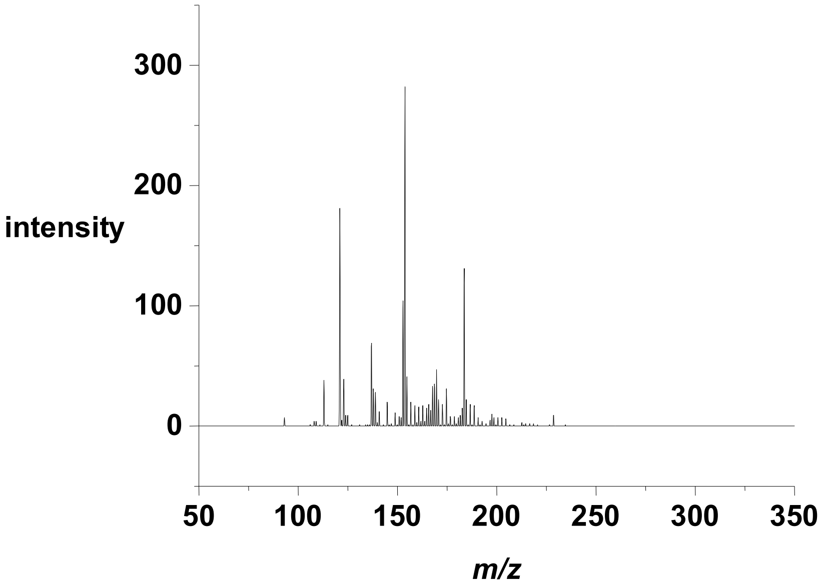


**Figure S22.** ESI-MS spectrum of benzyl alcohol after 5 min of plasma treatment in the presence of benzoic acid

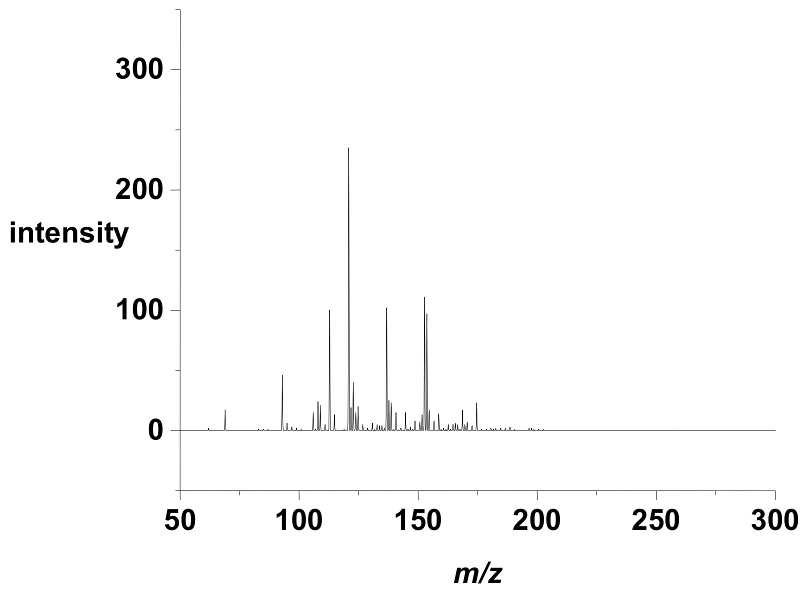


**Figure S23.** ESI-MS spectrum of benzaldehyde after 5 min of plasma treatment in the presence of benzoic acid

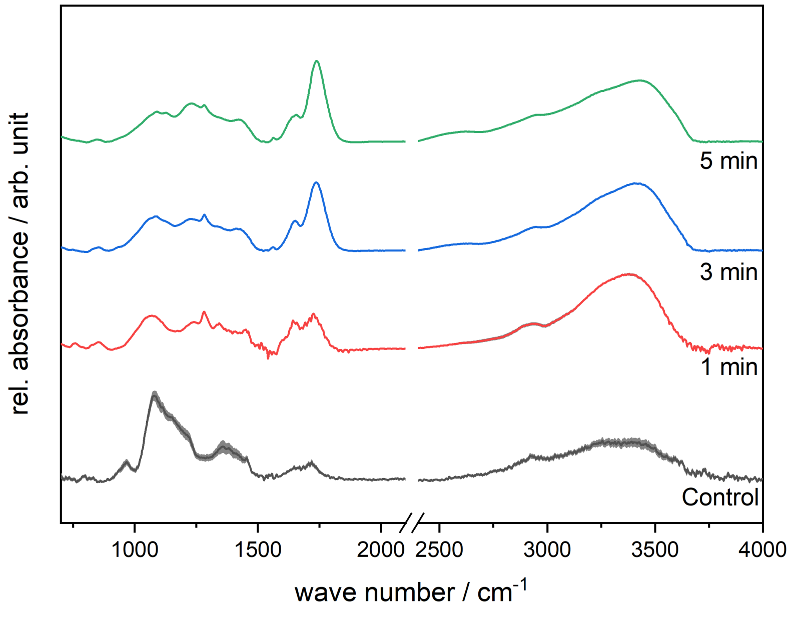


**Figure S24.** Mean FTIR-spectra of plasma-treated benzyl alcohol in the range of 700-1900 $cm^{-1}$ as a function of different treatment times. Standard deviation of the mean is shown as grey area at each graph.

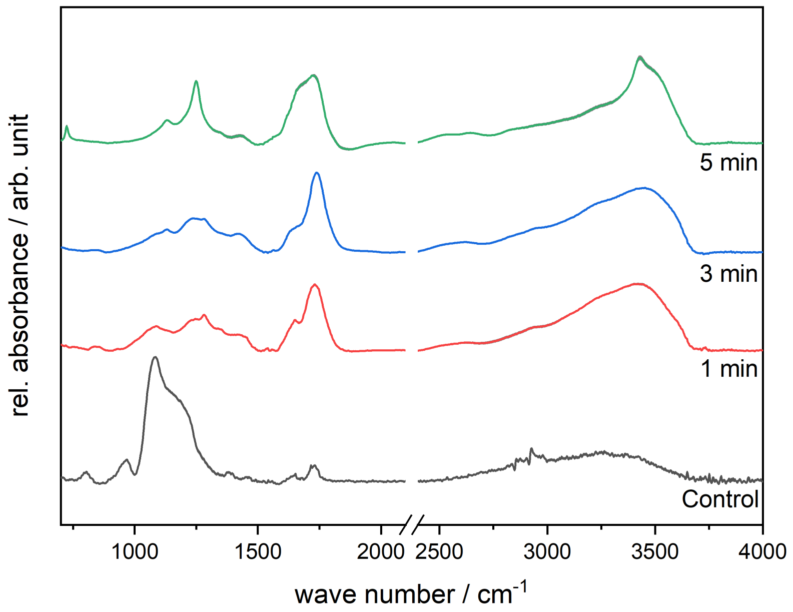


**Figure S25.** Mean FTIR-spectra of plasma-treated benzyl aldehyde in the range of 700-1900 $cm^{-1}$ as a function of different treatment times.

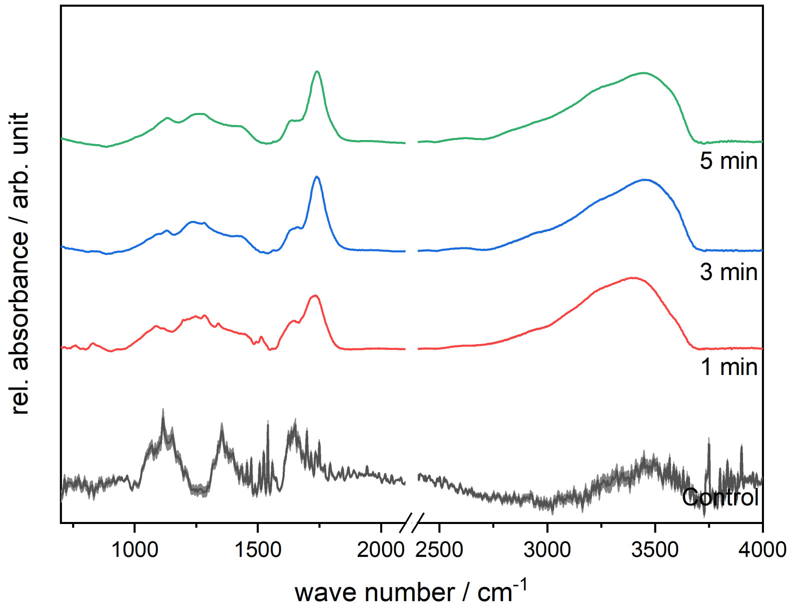


**Figure S26.** Mean FTIR-spectra of plasma-treated phenol in the range of 700-1900 $cm^{-1}$ as a function of different treatment times.

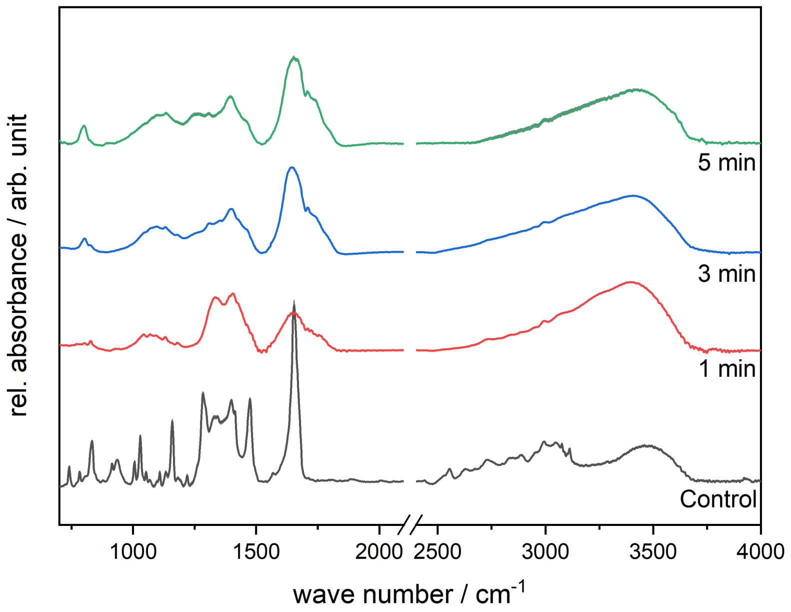


**Figure S27.** Mean FTIR-spectra of plasma-treated benzyl alcohol in the presence of complex **A** in the range of 700-1900 $cm^{-1}$ as a function of different treatment times.

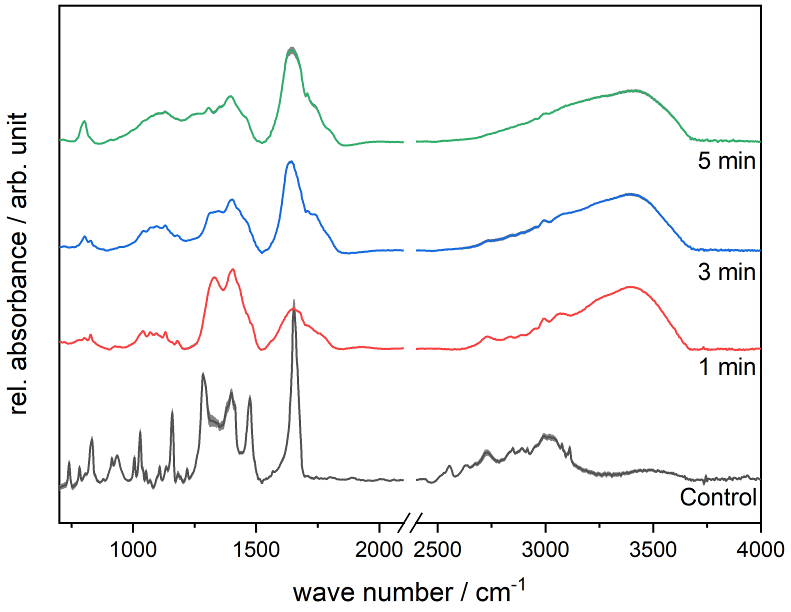


**Figure S28.** Mean FTIR-spectra of plasma-treated benzyl aldehyde in the presence of complex **A** in the range of 700-1900 $cm^{-1}$ as a function of different treatment times.

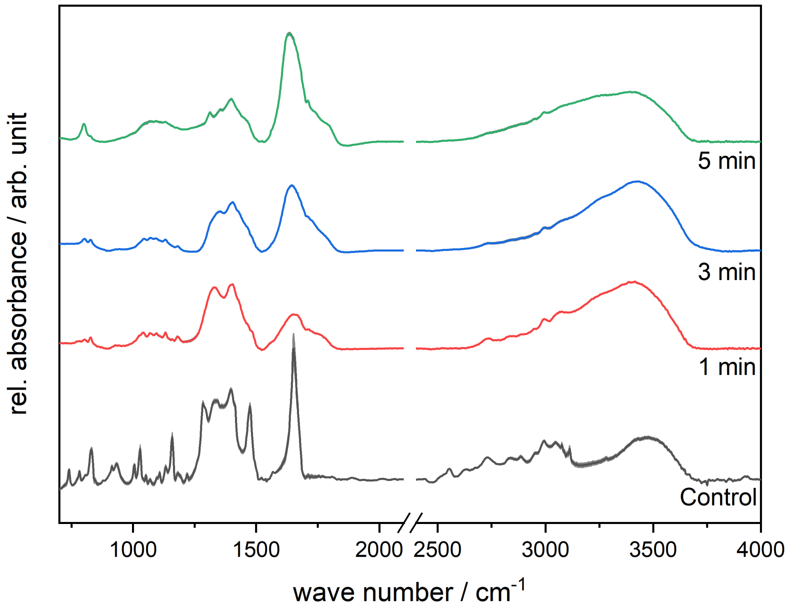


**Figure S29.** Mean FTIR-spectra of plasma-treated phenol in the presence of complex **A** in the range of 700-1900 cm$^{-1}$ as a function of different treatment times.

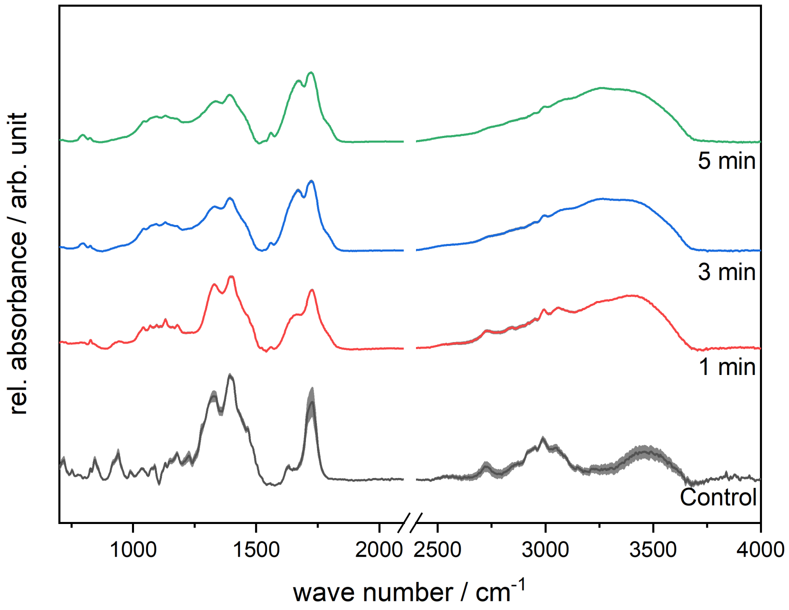


**Figure S30.** Mean FTIR-spectra of plasma-treated benzyl alcohol in the presence of complex **B** in the range of 700-1900 cm$^{-1}$ as a function of different treatment times.

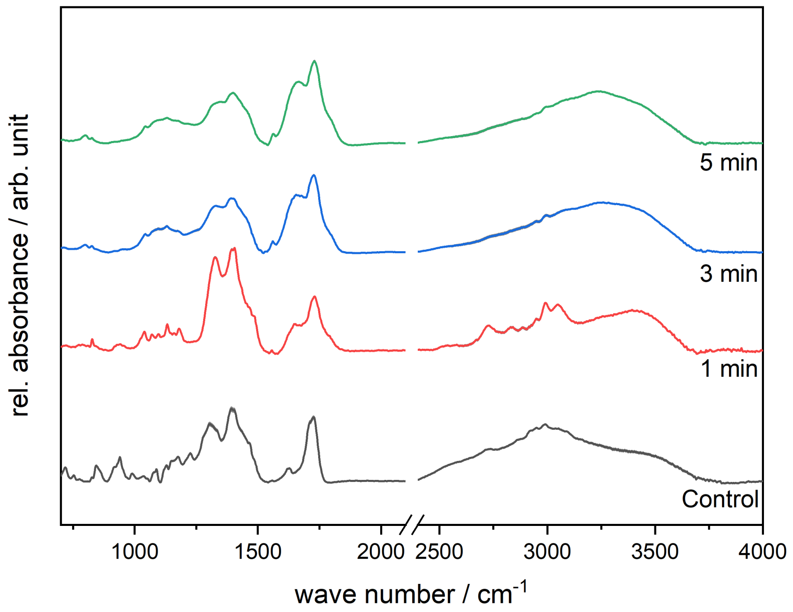


**Figure S31.** Mean FTIR-spectra of plasma-treated benzyl aldehyde in the presence of complex **B** in the range of 700-1900 $cm^{-1}$ as a function of different treatment times.

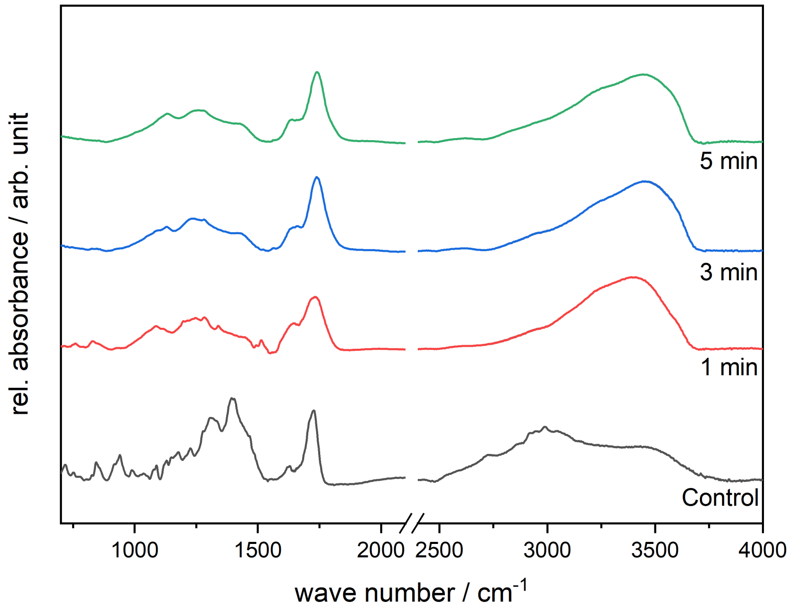


**Figure S32.** Mean FTIR-spectra of plasma-treated phenol in the presence of complex **B** in the range of 700-1900 $cm^{-1}$ as a function of different treatment times.

1. Kogelheide, F.; Kartaschew, K.; Strack, M.; Baldus, S.; Metzler-Nolte, N.; Havenith, M.; Awakowicz, P.; Stapelmann, K.; Lackmann, J.-W., FTIR spectroscopy of cysteine as a ready-to-use method for the investigation of plasma-induced chemical modifications of macromolecules. *Journal of Physics D: Applied Physics* **2016,** *49* (8), 084004.
